# A Highly Sensitive Dual-Signal Strategy via Inner Filter Effect between Tween 20-Gold Nanoparticles and CdSe/ZnS Quantum Dots for Detecting Cu^2+^

**DOI:** 10.3390/mi14050902

**Published:** 2023-04-23

**Authors:** Yong Xie, Chao Bian, Mingjie Han, Ri Wang, Yang Li, Yuhao Xu, Shanhong Xia

**Affiliations:** 1 State Key Laboratory of Transducer Technology, Aerospace Information Research Institute, Chinese Academy of Sciences, Beijing 100190, China; 2 School of Electronic, Electrical and Communication Engineering, University of Chinese Academy of Sciences, Beijing 100049, China

**Keywords:** fluorescence, colorimetry, copper, CdSe/ZnS quantum dots, gold nanoparticles, inner filter effect

## Abstract

A highly sensitive and accurate dual-signal strategy is developed for trace Cu^2+^ detection based on the inner filter effect (IFE) between Tween 20-gold nanoparticles (AuNPs) and CdSe/ZnS quantum dots (QDs). Tween 20-AuNPs are utilized as colorimetric probes and excellent fluorescent absorbers. The fluorescence of CdSe/ZnS QDs can be quenched efficiently by Tween 20-AuNPs via IFE. In the presence of D-penicillamine, D-penicillamine induces the aggregation of Tween 20-AuNPs and the fluorescent recovery of CdSe/ZnS QDs at high ionic strength. Upon addition of Cu^2+^, D-penicillamine tends to selectively chelate with Cu^2+^ and then forms the mixed-valence complexes, which consequently inhibits the aggregation of Tween 20-AuNPs and the fluorescent recovery. The dual-signal method is used to quantitatively detect trace Cu^2+^, with low detection limits of 0.57 μg/L and 0.36 μg/L for colorimetry and fluorescence, respectively. In addition, the proposed method using a portable spectrometer is applied to the detection of Cu^2+^ in water. This sensitive, accurate and miniature sensing system has potential in environmental evaluations.

## 1. Introduction

Heavy metal pollution is a highly concerning environmental issue due to its high toxicity, environmental persistence, and non-biodegradability [1]. Cu^2+^ is a major component of heavy metal pollution with its widespread application in agriculture and industry. However, Cu^2+^ has an important impact on various physiological processes, such as redox reaction, electron transport and prooxidant action [2]. Excessive Cu^2+^ is highly toxic and pathogenic to organisms, leading to serious nervous system diseases, such as Alzheimer’s disease and Parkinson’s disease [3]. Hence, developing valid methods for highly selective and sensitive detection of Cu^2+^ has great significance.

Numerous analytical approaches have been employed for detecting Cu^2+^, including colorimetry [4], fluorometry [5], surface-enhanced Raman spectroscopy [6], and inductively coupled plasma mass spectrometry (ICP-MS) [7], etc. Fluorometry is famous for its highly sensitive quantification, and colorimetry is favored for its simple, cost-effective, and naked-eye readout [8]. The dual-signal strategy takes advantage of fluorimetric and colorimetric methods, which can not only realize the highly sensitive and visual detection of Cu^2+^ conveniently but also enhance reliability and robustness by multiple output signals [9]. In general, the dual-signal strategy can contribute to enhancing application flexibility and detection accuracy, thus making the detected results more convincing.

AuNPs are especially attractive as a part of colorimetric or fluorescent sensors by reason of their superior optical properties such as adjustable absorption band, high extinction coefficient and color changes of solution [10]. AuNPs display an absorption band when electromagnetic radiation induces the collective oscillation of conduction electrons, which is referred to as the localized surface plasmon resonance (LSPR). Typically, LSPR is mainly dependent on the shape, size and inter-particle spacing of the nanoparticles [11]. Recently, colorimetric sensors based on the LSPR of AuNPs have been developed for the detection of pathogenic bacteria [12], enzymes [13], and thallium [14]. Quantum dots (QDs) have become a research hotspot because of their excellent photophysical properties, for example, tunable and narrow emission spectrum, high quantum yield, and high stability and photobleaching threshold [15]. Benefiting from these properties, QDs have been widely used as photocatalysts [16,17], photoelectric devices [18], biological imaging [19] and fluorescent probes [20]. Due to the excellent properties of QDs and the high extinction coefficient of AuNPs, various novel fluorescence sensors are composed of AuNPs and QDs, which are developed for the detection of thiourea [20], cysteine [21], and organophosphorus pesticide [22]. Most of them are based on fluorescence resonance energy transfer (FRET), in which QDs are considered to be ideal fluorescent donors, and AuNPs are outstanding absorbers. However, the FRET process has to consider the long-range dipole–dipole interactions, leading to a strict distance requirement of 1–10 nm between the QDs and AuNPs [23]. Therefore, the FRET-based sensors always require complicated chemical modification on the surface of QDs or AuNPs, which causes the detection procedure to be very complex and time-consuming. In contrast with the FRET process, the inner filter effect (IFE) process does not require close proximity between QDs or AuNPs, which offers a flexible and simple method to design the fluorescence probes [24]. Recently fluorescence probes based on IFE have been widely studied, such as for the detection of T-2 toxin [25], profenofos [26], sulfamethazine [27], and melamine [28]. The mechanism of IFE refers to the absorber absorbs the emission and/or excitation light of the fluorophore, resulting in the fluorescence quenching of the fluorophore [29]. For a competitive performance of the IFE-based sensors, the absorption spectra of absorbers should sufficiently overlap the emission and/or excitation spectra of fluorophores, which indicates absorber can effectively tune the fluorescence intensity of fluorophore [30]. Thus, the absorbance change of absorber will be efficiently converted to the fluorescence signal with an exponential change, which brings a higher sensitivity for detection according to the corrected fluorescence intensity equation by Albinsson [31].

**Figure 1:**
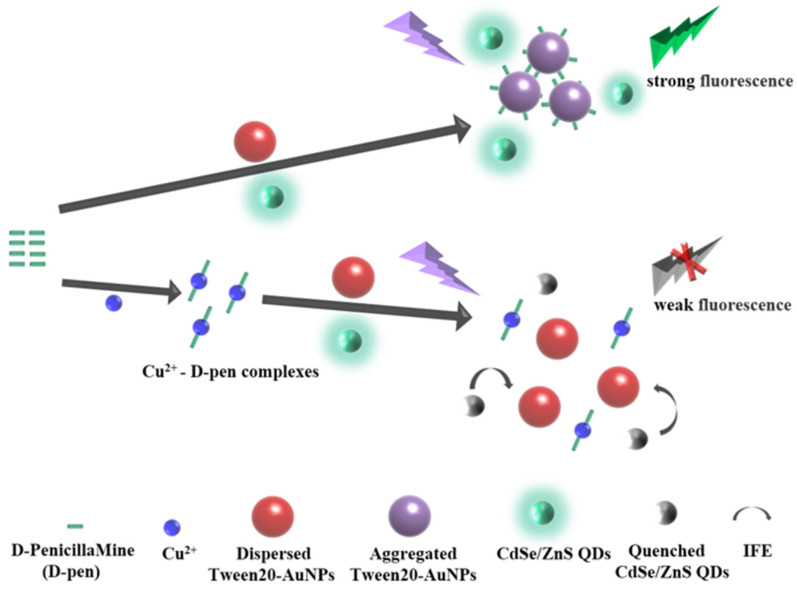
Schematic diagram of the dual-signal sensing system for Cu^2+^.

In this work, a dual-signal sensing system based on colorimetry and fluorescence is constructed to detect trace Cu^2+^ via the IFE process between Tween 20-AuNPs and CdSe/ZnS QDs. Significantly, D-penicillamine is used as an aggregation agent, which can induce the aggregation of Tween 20-AuNPs and selectively chelate with Cu^2+^ [32]. Tween 20 can keep AuNPs stable at higher ionic strength and play an important role in detecting trace Cu^2+^. The principle of the dual-signal sensing system is clarified in Figure 1. In the presence of D-penicillamine, D-penicillamine can cause the aggregation of Tween 20-AuNPs, and then CdSe/ZnS QDs exhibit a strong fluorescence emission, followed by the solution color change from red to dark purple. When Cu^2+^ is introduced, D-penicillamine prefers to chelate with Cu^2+^ and then forms the mixed-valence complexes, which consequently inhibits the aggregated state of Tween 20-AuNPs and decreases the fluorescence emission of CdSe/Zns QDs because of the IFE process. In general, the absorbance of Tween 20-AuNPs and the fluorescence intensity of CdSe/ZnS QDs depend on Cu^2+^ concentration. With the addition of Cu^2+^, the absorbance of Tween 20-AuNPs increases, whereas the fluorescence intensity of CdSe/ZnS QDs decreases. This method using a portable spectrometer, is successfully applied to the detection of Cu^2+^ in a real sample. Therefore, a highly sensitive and accurate dual-signal sensing system for trace Cu^2+^ detection has been developed.

## 2. Materials and Methods

### 2.1. Materials and Apparatus

Na_2_HPO_4_, NaH_2_PO_4_, and HAuCl_4_·4H_2_O from Sinopharm Chemical Reagent Co., Ltd. (Shanghai, China), NaCl from Aladdin Chemistry Co., Ltd. (Shanghai, China), and Trisodium citrate from Sigma–Aldrich (St. Louis, MO, USA). CdSe/ZnS QDs from Beijing Beidajubang Science & Technology Co., Ltd. (Beijing, China), Tween 20 from Sangon Biotech Co., Ltd. (Shanghai, China), and D-penicillamine from Macklin Biochemical Co., Ltd. (Shanghai, China). The deionized water produced by Millipore DQ3UV (Millipore Company, MA, USA).

The absorption spectra and fluorescent spectra were measured by the spectrophotometer (QE65pro), the UV-VIS-NIR light source was DH-mini, and the excitation light source was LED405 (Ocean Optics, Dunedin, FL, USA). Transmission electron microscopy (TEM) image was measured by Tecnai G2 F30 (FEI Company, Hillsboro, OR, USA). Zeta potential and dynamic light scattering (DLS) were measured by Zetasizer Nano ZS90 (Malvern Instruments, Malvern, UK). The fluorescence lifetime measurement was measured using the FLS980 spectrometer (Edinburgh Instruments, Edinburgh, UK). 

### 2.2. Synthesis of AuNPs

The trisodium citrate reduction method was used to synthesize AuNPs [33]. Briefly, 2 mL HAuCl_4_ solution (1%, *w*/*v*) was added to deionized water (98 mL) and then heated to boiling with strong stirring. After that, a 2.3 mL trisodium citrate solution (1%, *w*/*v*) was quickly added to the prepared solution. The mixture was further stirred and boiled for 15 min. Finally, the mixed solution was cooled to room temperature. Tween 20-AuNPs solution was formed by mixing 10 mL of Tween 20 (0.01%, *v*/*v*) with 10 mL of AuNPs. In addition, all glassware was soaked in aqua regia for 24 h and rinsed with deionized water. 

### 2.3. Colorimetric and Fluorometric Detection for Cu^2+^

In order to detect Cu^2+^, D-penicillamine (100 μL) and different concentrations of 100 μL Cu^2+^ were incubated at 30 °C for 30 min. Next, NaCl solution (30 μL), phosphate buffer (20 μL, 200 mM), and deionized water (500 μL) were added to the above mixture. After that, Tween 20-AuNPs (200 μL, 0.429 nM) was added to the mixed solution, which was incubated at room temperature. Finally, CdSe/ZnS QDs (50 μL, 20 nM) was added to the prepared solution, and then the fluorescence spectra and absorption spectra were recorded, respectively. Several experimental parameters, such as the concentrations of Tween 20, NaCl, and D-penicillamine, the pH value of the buffer solution, and the incubation time, were optimized.

## 3. Results and Discussion

### 3.1. Characterization of Tween 20-AuNPs and CdSe/ZnS QDs

**Figure 2:**
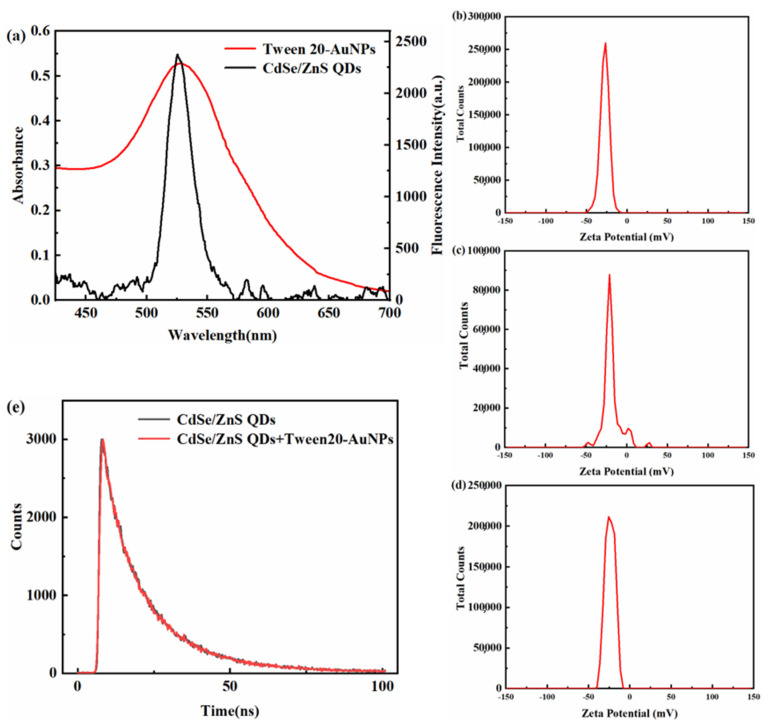
(**a**) Absorption spectrum of Tween 20-AuNPs and fluorescence spectrum of CdSe/ZnS QDs. (**b**–**d**) Zeta potential of AuNPs, Tween 20-AuNPs, and CdSe/ZnS QDs. (**e**) Fluorescence lifetimes of CdSe/ZnS QDs with and without Tween 20-AuNPs.

Tween 20-AuNPs and CdSe/ZnS QDs are considered to be the main components of the dual-signal strategy. The spectral overlap and optical properties of these two materials will directly influence the quenching efficiency of the dual-signal sensing system. Tween 20-AuNPs are considered to be highly efficient absorbers by reason of their high extinction coefficient. CdSe/ZnS QDs have excellent photostability, water dispersion and a narrow emission spectrum with the highest emission at 525 nm. Figure 2a shows the absorption spectrum of Tween 20-AuNPs and the fluorescence spectrum of CdSe/ZnS QDs. As shown in Figure 2a, Tween 20-AuNPs exhibit an absorption peak at 528 nm. It can be found that the absorption spectrum can overlap the fluorescence emission spectrum in a wide range, which provides a necessary condition for the generation of IFE. Hence, the fluorescence intensity of CdSe/ZnS QDs is remarkably decreased if these two materials coexist. 

**Figure 3:**
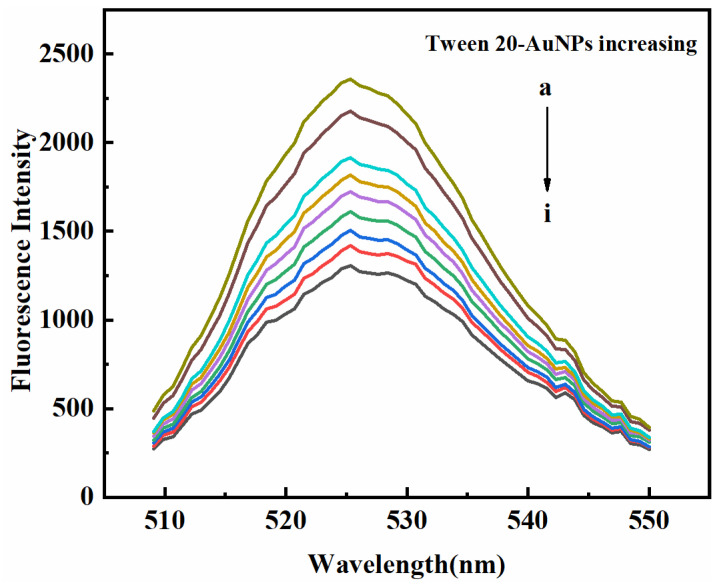
Fluorescence spectra of CdSe/ZnS QDs with different concentrations of Tween 20-AuNPs. The final concentrations of Tween 20-AuNPs in samples (a)–(i) are 0, 1.72, 3.46, 4.29, 5.15, 6.01, 6.86, 7.72, and 8.58 × 10^−11^ M, respectively. CdSe/ZnS QDs, 1 × 10^−9^ M.

As mentioned earlier, AuNPs are synthesized using the trisodium citrate reduction method, resulting in AuNPs being capped with negative citrate ions. Thus, as shown in Figure 2b,c, AuNPs and Tween 20-AuNPs, both possess negative charges, which are demonstrated through the zeta potential measurements of AuNPs (−27.5 mV) and Tween 20-AuNPs (−20.4 mV). In addition, the zeta potential of CdSe/ZnS QDs is −23.9 mV (Figure 2d). Guo et al. demonstrated that negatively charged AuNPs and positively charged QDs might generate the FRET donor–acceptor systems via electrostatic attractive interactions due to the electrostatic attractive interactions effectively shortening the gap between AuNPs and QDs [34]. Hence, there may be no electrostatic interaction and FRET between CdSe/ZnS QDs and Tween 20-AuNPs, due to they both possess negative charges. In addition, in the absence and presence of Tween 20-AuNPs, we measured the fluorescence lifetime of CdSe/ZnS QDs. Figure 2e shows that the average lifetime of CdSe/ZnS QDs has no remarkable difference in the presence of Tween 20-AuNPs, which proves that there is no energy transfer between Tween 20-AuNPs and CdSe/ZnS QDs, due to the fluorescence lifetime could be significantly changed by FRET but is unaffected by IFE [35]. The above results exhibit the remarkable fluorescence decrease of CdSe/ZnS QDs ascribed to the IFE process. The fluorescence spectra of CdSe/ZnS QDs mixed with various concentrations of Tween 20-AuNPs were recorded to prove the IFE-based sensing system. Figure 3 shows that with increasing the concentrations of Tween 20-AuNPs, the fluorescence of CdSe/ZnS QDs decreases gradually. Therefore, the absorbance of Tween 20-AuNPs can effectively tune the fluorescence of CdSe/ZnS QDs at 525 nm through IFE.

### 3.2. Dual-Signal Mechanism for Cu^2+^ Assay

**Figure 4:**
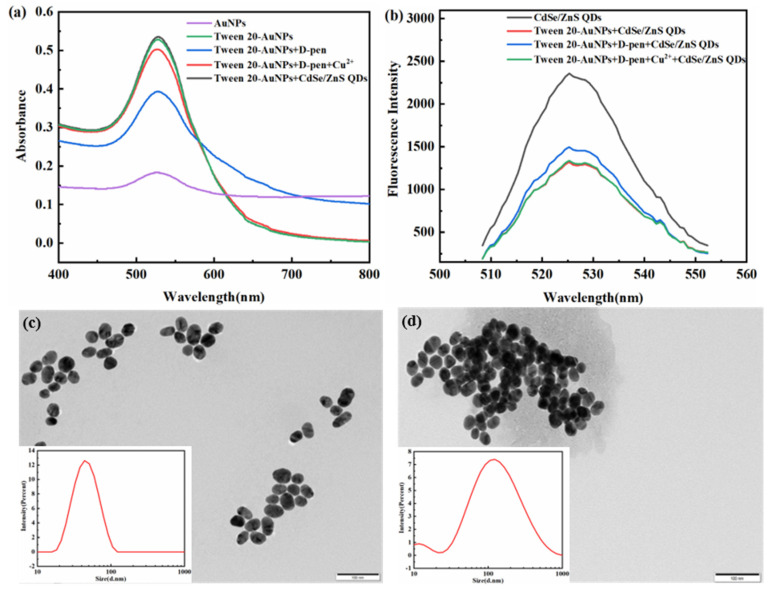
(**a**) Absorption spectra and (**b**) fluorescence spectra of the dual-signal sensing system. TEM images of Tween 20-AuNPs in the (**c**) absence and (**d**) presence of D-penicillamine(D-pen). Inset: DLS of Tween 20-AuNPs. The final concentrations of testing reagents were as follows: AuNPs, 8.58 × 10^−11^ M; Tween 20-AuNPs, 8.58 × 10^−11^ M; D-penicillamine, 4 μM; CdSe/ZnS QDs, 1 nM; and NaCl, 30 mM. Cu^2+^, 10 μg/L.

D-penicillamine is a chelator for copper contamination. It not only shows high affinity to the surface of Tween 20-AuNPs through its thiol groups but can also induce the aggregation of Tween 20-AuNPs at high ionic strength via electrostatic interaction or hydrogen bonding [32]. After the addition of D-penicillamine, Cu^2+^ is reduced to Cu^+^, and then they form complexes such as (Cu(II)_6_Cu(I)_8_ (D-penicillamine)_12_ Cl)^5−^ through the thiol groups of D-penicillamine, which effectively inhibits the aggregated state of Tween 20-AuNPs by reason of the stronger chelation between D-penicillamine and Cu^2+^ [36]. To prove the feasibility of dual-signal strategy for Cu^2+^ assay, the anti-aggregation mechanism of Tween 20-AuNPs was studied by absorption spectra, fluorescence spectra, and TEM (Figure 4).

As shown in Figure 4a, AuNPs and Tween 20-AuNPs exhibit absorption peak at 526 nm and 528 nm, respectively. It is found that AuNPs (Figure 4a) are aggregated at such high ionic strength with a lower absorbance, but Tween 20-AuNPs are well-dispersed with a higher absorbance due to Tween 20 can modify AuNPs to prevent the aggregation of AuNPs at high ionic strength. This shows that pure AuNPs are easy to aggregate even in the absence of D-penicillamine at high ionic strength, which illustrates that pure AuNPs are not suitable for dual-signal sensing systems. The introduction of D-penicillamine can induce the aggregation of Tween 20-AuNPs and decrease their absorbance, along with the color of the solution changing from red to dark purple. Upon addition of Cu^2+^, D-penicillamine tends to form compounds with Cu^2+^, and then Tween 20-AuNPs remain dispersed with the absorption peak at 528 nm. These results illustrate the feasibility of detecting Cu^2+^ through the colorimetric response of Tween 20-AuNPs. In addition, the absorption spectrum of Tween 20-AuNPs shows no marked distinction in the absence and presence of CdSe/ZnS QDs, illustrating that molecular interaction between Tween 20-AuNPs and CdSe/ZnS QDs is basically non-existent. 

Fluorescence spectra also demonstrate that Cu^2+^ can inhibit the aggregation of Tween 20-AuNPs. Figure 4b shows that CdSe/ZnS QDs exhibit a strong fluorescent signal at about 525 nm, but the fluorescence of CdSe/ZnS QDs is immediately quenched after mixing with Tween 20-AuNPs via IFE. After the addition of D-penicillamine, as mentioned above, the absorbance of Tween 20-AuNPs decreases significantly, and then CdSe/ZnS QDs can recover the quenched fluorescence. However, when Cu^2+^ is firstly incubated with D-penicillamine, the absorbance of Tween 20-AuNPs is recovered, and the fluorescence recovery of CdSe/ZnS QDs is prevented, which directly proves the feasibility of dual-signal strategy for Cu^2+^ detection. Moreover, TEM images (Figure 4c,d) and DLS results (inset of Figure 4c,d) clearly exhibit that morphology changes of Tween 20-AuNPs in the absence and presence of D-penicillamine, just as the D-penicillamine can induce the aggregated state of Tween 20-AuNPs (Figure 4d), which are in agreement with the previous optical spectra.

### 3.3. Optimization of Experimental Conditions for Cu^2+^ Detection

Several key parameters affecting the dual-signal assay for Cu^2+^ were optimized, such as the concentrations of Tween 20, NaCl, and D-penicillamine, the incubation time and the pH value.

**Figure 5:**
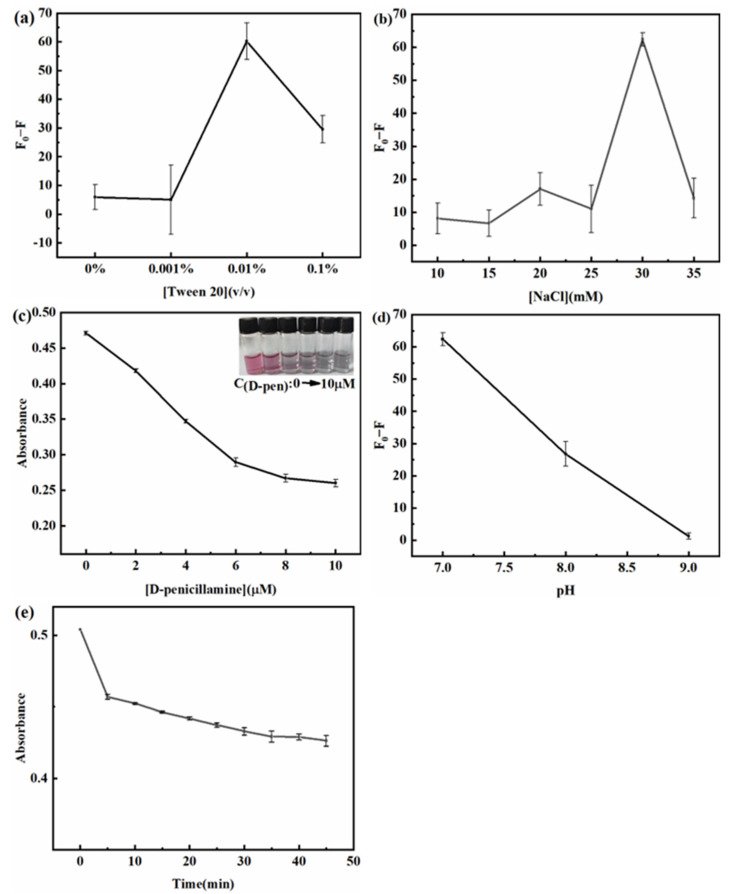
Effect of (**a**) Tween 20 (*v*/*v*), (**b**) NaCl concentration, and (**d**) pH on the fluorescence intensity (F_0_ − F) of the sensing system. (**c**) The absorbance of Tween 20-AuNPs versus the concentration of D-penicillamine. (**e**) Effect of the incubation time on the absorbance of Tween 20-AuNPs. The final concentrations of testing reagents were as follows: AuNPs, 8.58 × 10^−11^ M; Tween 20-AuNPs, 8.58 × 10^−11^ M; CdSe/ZnS QDs, 1 nM. Cu^2+^, 4 μg/L.

Tween 20 can be used as a nonionic surfactant, whose function is to stabilize AuNPs at high ionic strengths and ensure that the aggregation of Tween 20-AuNPs is caused by D-penicillamine. Significantly, pure AuNPs are easy to aggregate even in the absence of D-penicillamine at high ionic strength. In this work, at higher ionic strength, it was proved that a low concentration of D-penicillamine would cause obvious aggregation of Tween 20-AuNPs (Figure 4a), which was extremely useful for detecting trace Cu^2+^. As shown in Figure 5a, it is found that 0.01% (*v*/*v*) Tween 20 shows the optimum response in various concentrations of Tween 20 (0%, 0.001%, 0.01% and 0.1%, *v*/*v*). While the addition of Tween 20 is less than 0.01% (*v*/*v*), the fluorescence intensity (F_0_ − F, where F_0_ and F are the fluorescence intensity of CdSe/ZnS QDs in the absence and presence of Cu^2+^, respectively) is weak, and Tween 20-AuNPs are aggregated because of Tween 20 is too few to maintain the stability of Tween 20-AuNPs in 30 mM NaCl solution. While the addition of Tween 20 is higher than 0.01% (*v*/*v*), although Tween 20-AuNPs have good stability in a high NaCl condition, there may be competitive adsorption between Tween 20 and D-penicillamine, leading to insensitive response for detecting trace Cu^2+^. Therefore, 0.01% (*v*/*v*) Tween 20 is chosen for further experiments.

The concentration of NaCl has an important influence on the Tween 20-AuNPs aggregation status due to NaCl can constrict the electrical double layer around the AuNPs surface [37]. From Figure 5b, while the final concentration of NaCl is less than 30 mM, the aggregation of Tween 20-AuNPs does not take place even in the presence of D-penicillamine, and the fluorescence intensity (F_0_ − F) has no obvious increase. Moreover, Tween 20-AuNPs are aggregated under excessive NaCl concentration (35 mM), even in the absence of D-penicillamine. Therefore, 30 mM is chosen as the optimal concentration of NaCl.

D-penicillamine is used as an aggregation agent of Tween 20-AuNPs and directly affects the linear range and sensitivity of the sensing system. To obtain ideal concentration of D-penicillamine, the aggregated state of Tween 20-AuNPs was investigated at various concentrations of D-penicillamine. Figure 5c shows that while the final concentration of D-penicillamine ranged from 0 μM to 4 μM, the change in absorbance is remarkable. While the final concentration of D-penicillamine is higher than 6 μM, the absorbance of Tween 20-AuNPs has no obvious change, which is insensitive to detect low concentrations of Cu^2+^. From the inset photograph in Figure 5c, with increasing the D-penicillamine concentration, the color of the solution changes from red to dark purple. However, when the final concentration of D-penicillamine is higher than 4 μM, the color change is not obvious. Therefore, in order to achieve higher sensitivity and a wider linear range, 4 μM D-penicillamine is selected as a suitable concentration for further experiments.

pH significantly affects the aggregation degree of Tween 20-AuNPs. In this system, the electrostatic interaction may be the dominant factor for the aggregated state of Tween 20-AuNPs. As reported in the literature, the zwitterionic form of D-penicillamine might enhance the electrostatic attractive interaction when the pH value is in the range of 3.0–7.0 [32,38]. According to the tests, when pH was less than 7.0 in the sensing system, it was found that Tween 20-AuNPs were extremely easy to aggregate even at low concentrations of D-penicillamine, but the state of aggregation was not stable. Therefore, pH should not be less than 7.0 for the stable aggregation of Tween 20-AuNPs. To obtain optimal pH, the fluorescence intensity (F_0_ − F) was studied in the pH range of 7.0–9.0. Figure 5d shows the maximum fluorescence intensity (F_0_ − F) obtained at pH 7.0 because D-penicillamine is in an anionic form when pH is higher than 7.0, then the aggregation degree of Tween 20-AuNPs decreases. Thus, pH 7.0 is chosen as the optimum pH for Cu^2+^ detection.

To obtain the optimal colorimetric and fluorescence response, the incubation time was investigated in the dual-signal sensing system. From Figure 5e, the result shows the aggregation of Tween 20-AuNPs is basically completed within 30 min, and then the absorbance of Tween 20-AuNPs is kept stable. Therefore, the incubation time is chosen as 30 min to ensure the aggregation of Tween 20-AuNPs is complete and stable. In addition, the fluorescence of CdSe/ZnS QDs is instantly quenched via IFE when CdSe/ZnS QDs are added into Tween 20-AuNPs, which results in no incubation after the addition of CdSe/ZnS QDs into the sensing system.

### 3.4. Dual-Signal Detection of Cu^2+^

**Figure 6:**
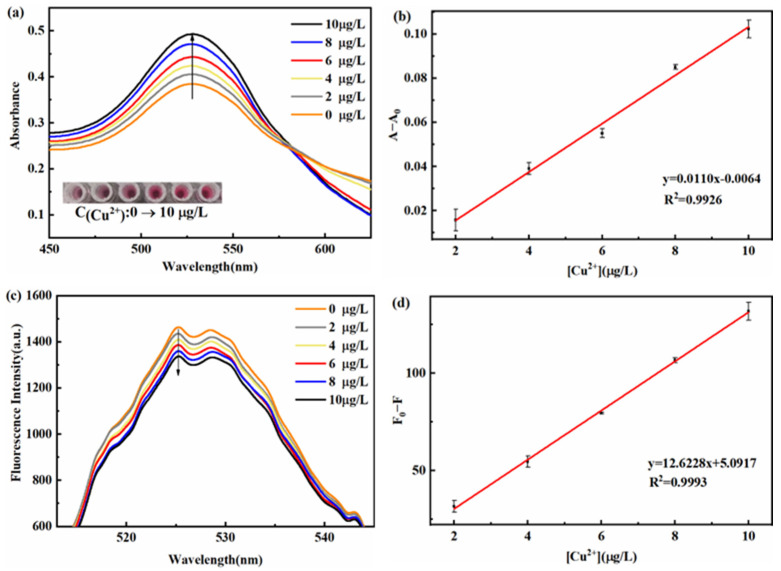
(**a**) Absorption spectra and (**c**) fluorescence spectra of the sensing system versus the concentration of Cu^2+^. Linear relationship of (**b**) the absorbance (A − A_0_) and (**d**) the fluorescence intensity (F_0_ − F) versus the concentration of Cu^2+^.

Under the optimal conditions, the absorbance and fluorescence intensity of the dual-signal sensing system was measured for Cu^2+^ detection. As shown in Figure 6a, the absorbance of Tween 20-AuNPs increases gradually with the increasing concentration of Cu^2+^, simultaneously accompanying the solution color changes from dark purple to red. Thus, the obvious color changes relating to Cu^2+^ concentration can be applied to the visual detection of Cu^2+^. There is a good linear relationship between the increased absorbance of Tween 20-AuNPs (A − A_0_, where A and A_0_ are the absorbances of Tween 20-AuNPs in the presence and absence of Cu^2+^, respectively) and the Cu^2+^ concentration ranging from 2 to 10 μg/L with R^2^ = 0.9926 (Figure 6b). The limit of detection (LOD) and the limit of quantification (LOQ) for Cu^2+^ obtained by the colorimetric method is calculated as 0.57 μg/L and 1.8 μg/L, respectively. LOD = 3σ/s, LOQ = 10σ/s, where σ is the standard deviation of a blank solution, and s is the slope of the calibration curve.

Meanwhile, the fluorescence recovery of CdSe/ZnS QDs is also based on the Cu^2+^ concentration. Figure 6c shows that the fluorescence intensity of CdSe/ZnS QDs gradually decreases with the increase of the Cu^2+^ content. As shown in Figure 6d, a good linear relationship between the Cu^2+^ concentration and the fluorescence intensity (F_0_ − F) is acquired in the range of 2~10 μg/L with R^2^ = 0.9993. The LOD and LOQ for Cu^2+^ obtained by the fluorescence method are 0.36 μg/L (3σ/s) and 1.2 μg/L (10σ/s), respectively. Furthermore, the proposed method is compared with other reported methods for determining Cu^2+^, as shown in Table 1. The proposed method is reliable and sensitive and has the potential to detect Cu^2+^ in practical applications.

### 3.5. Selectivity

**Figure 7:**
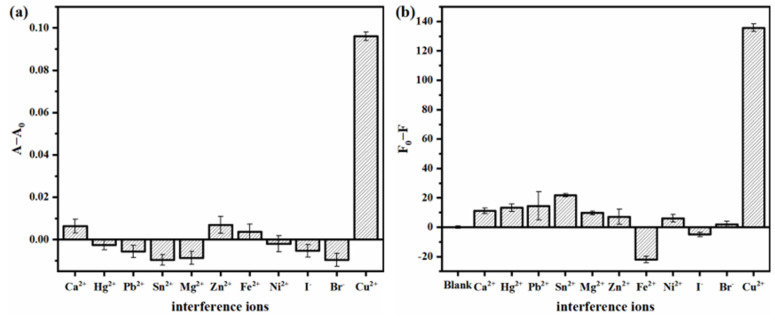
(**a**) Colorimetric and (**b**) fluorescent selectivity of the sensing system. (Cu^2+^, 10 μg/L; other interferences, 100 μg/L).

The possible interference of other ions was studied using 10-fold Ca^2+^, Hg^2+^, Pb^2+^, Sn^2+^, Mg^2+^, Zn^2+^, Fe^2+^, Ni^2+^, I^-^ and Br^-^ to estimate the selectivity of this system. From Figure 7, the maximum fluorescence response of Sn^2+^ and Fe^2+^ (except for Cu^2+^) is not higher than 16% (Figure 7b), the colorimetric (Figure 7a) and fluorescence (Figure 7b) response of most ions is less than 10% when the Cu^2+^ response is set to 100%. These results indicate that the sensing system possesses a good selective response toward Cu^2+^ because Cu^2+^ can selectively chelate with D-penicillamine. 

### 3.6. Determination of Cu^2+^ in Real Sample

The tap water sample has been detected by using ICP-MS in Pony Testing International Group. The concentration of Cu^2+^ was 1.44 μg/L, which was outside the linear range of 2~10 μg/L of this developed method. The standard addition method was used to investigate the applicability of a dual-signal sensing system for a Cu^2+^ assay. As shown in Table 2, the recoveries are in the range of 91.1% to 99.0% and from 85.6% to 100.2% for fluorescence and colorimetric detection, respectively. It can be seen that the recoveries inside the linear range are better than that outside the linear range. The recoveries of the developed methods in the detectable range are from 97.1% to 100.2%. According to the detected value within the linear range, the original concentration of Cu^2+^ in the tap water sample is calculated as 1.34 μg/L by using the standard addition method, which is in good agreement with the results obtained by ICP-MS (1.44 μg/L). This proves that the sensing system has potential in environmental evaluations.

## 4. Conclusions

In summary, a novel dual-signal sensing system based on Tween 20-AuNPs and CdSe/ZnS QDs is constructed for trace Cu^2+^ detection. The detection mechanism is characterized by absorption spectra, fluorescence spectra, TEM, fluorescence lifetime and zeta potential. Tween 20 can keep AuNPs stable at high ionic strength and plays an important role in detecting trace Cu^2+^. Tween 20-AuNPs have the dual functions of colorimetric probes and fluorescence absorbers in the sensing system. The fluorescence of CdSe/ZnS QDs decreases immediately via IFE when CdSe/ZnS QDs are added to Tween 20-AuNPs. D-penicillamine can effectively induce the aggregated state of Tween 20-AuNPs by decreasing their absorbance, which results in the recovery of IFE-quenched fluorescence of CdSe/ZnS QDs. With increasing Cu^2+^ concentration, the absorbance of Tween 20-AuNPs increases gradually, and the fluorescence of CdSe/ZnS QDs decreases gradually by reason of selective chelation between D-penicillamine and Cu^2+^. This sensing system using a portable spectrometer is applied to the detection of Cu^2+^ in water. The dual-signal sensing system exhibits high selectivity, sensitivity, and accuracy and has the potential to realize on-site detection in water environment evaluation.

## Figures and Tables

**Table 1:** Comparison of different methods for Cu^2+^ determination.

Probes	Methods	Linear Range	Detection Limit	Ref
plasmonic sugar nanoprobes	Colorimetric	0.001–100 mM	9.7 μM	[39]
gold nanorods	Colorimetric	0.05–4.0 μM	34 nM	[40]
silver nanocubes	Colorimetric	0.01–40 μM	10 nM	[41]
silver nanoclusters (NCs)	Fluorometric	0.1–200 μM	29 nM	[42]
AuNCs/CDs@SiO_2_	Fluorometric	0.5–16 μM	250 nM	[43]
SQDs/CQDs	Fluorometric	0.1–5.0 μM	31 nM	[44]
	Colorimetric	0.1–5.0 μM	47 nM	
CdSe/ZnS QDs/Tween 20-AuNPs	Fluorometric	2–10 μg/L (31–157 nM)	0.36 μg/L (5.7 nM)	This work
	Colorimetric	2–10 μg/L (31–157 nM)	0.57 μg/L (9.0 nM)	

**Table 2:** Determination of Cu^2+^ in tap water.

Tap Water (μg/L)	Added (μg/L)	Colorimetric Detection			Fluorescence Detection		
		Detected (μg/L)	Recovery (%)	RSD (%)	Detected (μg/L)	Recovery (%)	RSD (%)
1.44	5.0	6.45	100.2	1.4	6.39	99.0	3.5
	7.0	8.24	97.1	0.6	8.29	97.9	2.4
	10.0	10.0	85.6	1.8	10.55	91.1	2.1

## Data Availability

The data that support the findings of this study are available from the corresponding authors upon reasonable request.
